# THE ASSOCIATION BETWEEN PERSON-CENTERED PLANNING AND PERSON-REPORTED OUTCOMES IN HOME- AND COMMUNITY-BASED SERVICES

**DOI:** 10.1093/geroni/igad104.0808

**Published:** 2023-12-21

**Authors:** Natalie Chong, Joseph Caldwell

**Affiliations:** The Heller School for Social Policy and Management at Brandeis University, Waltham, Massachusetts, United States; Lurie Institute For Disability Policy, Brandeis University, Waltham, Massachusetts, United States

## Abstract

Person-centered planning (PCP) allows home and community-based service (HCBS) users to plan and coordinate services and supports according to their preferences and needs. The extent to which HCBS systems provide high-quality PCP and evidence for the relationship between PCP and user outcomes is limited. This study examines HCBS user characteristics related to PCP and the association between PCP and both unmet need and community living. We used the 2018-2019 National Core Indicators-Aging and Disability survey’s Person-Centered Planning Module, collected among adult Medicaid HCBS users in 12 states (n = 6,924). Independent variables included two general PCP measures (one on decision-making and another on preferences and choices being reflected in service plans) and a PCP fidelity scale to assess the extent to which users’ most recent service planning meeting was person-centered. Outcomes included two measures of unmet need for services and three measures of community living (i.e., participation, control, and satisfaction). We examined user characteristics associated with PCP and used logistic regression models to assess the relationship between PCP and outcomes. About 85% of users reported they were fully involved in decision-making, 77% reported their preferences and choices were reflected in their service plan, and 57% had their most recent service planning meeting coded as “high fidelity” on the PCP fidelity scale. Each marker of high-quality PCP was consistently related to lower likelihood of unmet need and greater likelihood of experiencing each community living outcomes. Findings suggest high-quality PCP is important for meeting HCBS users’ needs and improving community living outcomes.

